# How Long Do Buildings Live: A Systematic Review and Meta-Analysis of Global Building Lifetimes

**DOI:** 10.1021/acs.est.6c06705

**Published:** 2026-09-08

**Authors:** Kamila Krych, Nils Dittrich, Daniel B. Müller, Stefanie Hellweg

**Affiliations:** † 27219 Institute of Environmental Engineering, ETH Zurich, Zurich 8093, Switzerland; ‡ Department of Energy and Process Engineering, Norwegian University of Science and Technology, Trondheim 7034, Norway

**Keywords:** service life, turnover time, material stock, material flow analysis, survival analysis, right censoring, carbon lock-in, lifetime extension

## Abstract

Building lifetime is a foundational parameter for modeling stock dynamics, informing environmental impact assessment and urban development, yet current lifetime assumptions frequently lack empirical grounding. Although well-established in medicine and demography, survival analysis concepts have not penetrated building stock research to the same extent. This study bridges this gap through a systematic review and meta-analysis of 258 median building lifetime estimates across 46 global studies. Central to our analysis is right-censoring, when a building’s demolition remains unobserved at the end of the observation period, which, if unaccounted for in estimation, leads to underestimated lifetimes. We systematize five estimation approaches based on data type and the capacity to account for right-censoring, describe the currently available empirical evidence, and apply a meta-regression to identify significant predictors of median lifetimes: world region, use class, structural material, and right-censoring bias, the latter underestimating building lifetimes by 50% (95% CI: 49–51%). Of the five estimation approaches identified, three inherently consider right-censoring, one inherently cannot correct for it, and one requires an active correction step that is frequently omitted. Our work identifies critical data gaps and offers targeted recommendations for users and producers of lifetime estimates to improve transparency, reproducibility, and methodological rigor.

## Graphical abstract

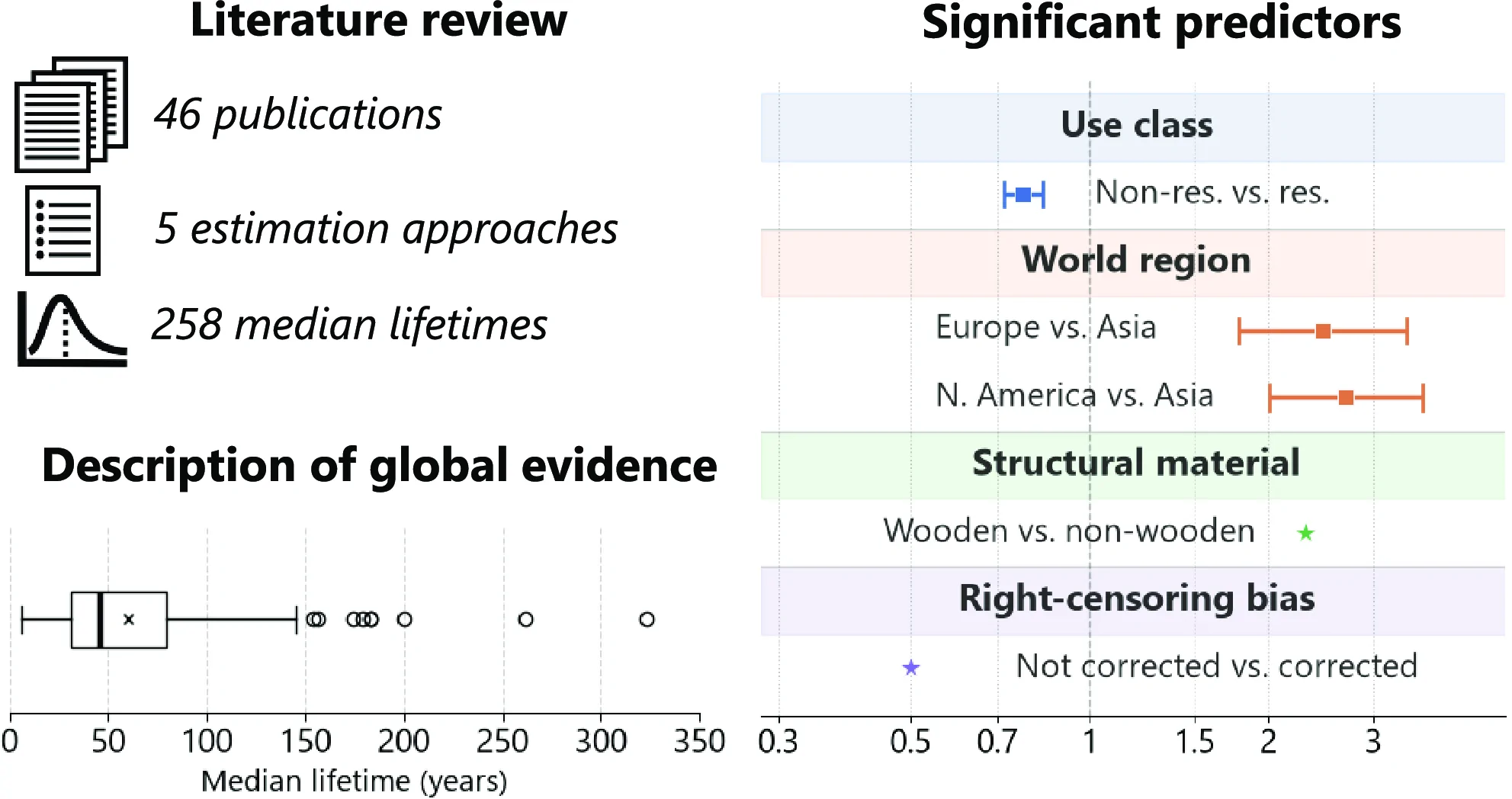

## 1 Introduction

Building lifetime is a critical parameter for understanding and mitigating the future environmental impacts of the building stock. It represents the temporal dimension in environmental assessment methods such as life cycle assessment (LCA) and dynamic stock modeling (DSM)—methods used to inform decisionmakers on the projected construction material use, demolition waste flows, energy use for operation, and others, including environmental trade-offs. All of these are highly sensitive to lifetime assumptions. The choice of building lifetime affects the annual embodied impacts, as well as recurring impacts associated with the replacement of building components. As a result, lifetime assumptions strongly affect the results of building LCAs, and this influence increases as buildings get more energy-efficient.^1,2^ In the DSM of buildings, even the shape of the lifetime distribution function (e.g., Weibull vs log-normal) significantly influences projected demolition waste.^3^ In addition, a good understanding of the lifetime expectancy is necessary to implement lifetime extension, which, as one of the pillars of the circular economy,^4^ would improve the circularity in the building sector. These examples clearly show that evidence-based knowledge on building lifetimes is essential for reliable prospective modeling of resource use and emissions trajectories.

However, lifetime assumptions are rarely based on empirical data. For example, in LCA, building lifetime is commonly assumed to be 50 years or another fixed value from 40 to 150 years, typically based on normative values or assumptions used in prior work.^5^ Initiatives to compile empirical lifetime data, such as the LiVES database established in 2010,^6^ have faced challenges in consolidating international evidence. Consequently, empirical data remains scattered, and a solid empirical basis for informed building lifetime assumptions is lacking.

Another pertinent issue is the methodological expertise involved in lifetime research. While lifetime distribution functions are broadly used in DSM,^7,8^ specialized statistical knowledge required to derive solid estimates is not native to the field. Lifetime and similar time-to-event data is the subject of survival analysis, a branch of statistics widely applied in disciplines such as medicine, demography, and reliability engineering.^9,10^ Survival analysis concepts have previously been an inspiration to DSM and broader industrial ecology research, for example, to map lifetime estimation approaches,^11,12^ model multidimensional lifetime patterns,^13^ or apply Kaplan–Meier estimators to buildings.^14^ Nonetheless, building stock research lags behind other fields of application, and state-of-the-art survival analysis techniques are currently absent from the field. As a result, existing taxonomies of lifetime estimation approaches^11,12^ do not consider methodological aspects of survival analysis which, when ignored, are known to introduce bias.^15^

The aim of this study is to investigate the data and methodological deficits in current building lifetime research by conducting a comprehensive review of empirical lifetime estimates of building stocks. Such a review is currently missing in the literature, even though reviews on other related topics have been performed, e.g., on built environment stock models,^16^ construction materials in DSM,^17^ building renovations in LCA,^18^ and life-cycle recurrent embodied energy in buildings.^19^ This paper seeks to (1) classify building lifetime estimation methodologies and corresponding methodological biases using survival analysis concepts; and (2) collect and assess available lifetime estimates to understand underlying variance patterns. Ultimately, this work intends to establish a robust empirical foundation for the field, identifying critical data gaps and offering practical guidance for future empirical studies and method development.

## 2 Methods

### 2.1 Definitions

Survival analysis is a field of research dedicated to the study of time-to-event data. In the context of this study, the event of interest is *building demolition*, which is the physical disappearance of the building, sometimes proxied by a demolition permit. Each building has a specific *realized lifetime* defined as the duration from its construction to demolition. Collectively, the lifetimes of individual buildings in a population are represented by a nonnegative random variable *T*, where the probability distribution of *T* characterizes the likelihood of a demolition as a function of age *t* across the stock. *Building mortality* refers to the hazard function representing the instantaneous risk of demolition occurring at a specific age *t*, conditional on the building having survived until that age.

Building lifetime can be quantified through two distinct analytical dimensions, which correspond to cohort and period life expectancy in demography.^20^ *Cohort lifetime measurement* uses a longitudinal approach to track the survival of a specific construction cohort over time. *Period lifetime measurement* uses a cross-sectional snapshot of a specific year to observe demolitions across all age groups simultaneously, creating a “synthetic cohort” representing a timely set of conditions. If the mortality rates are constant over time, the two approaches provide the same result.

The necessity of survival analysis arises from its ability to handle censoring, a condition where the exact event time (demolitions) for certain individuals is not fully observed.^9,10^ The most prevalent form is *right-censoring*, which occurs when buildings remain standing at the end of the observation period, leaving their demolition dates unknown. Right-censoring can be taken into account using survival analysis tools such as the Kaplan–Meier estimator.^15^ Failing to account for right-censoring, for example by omitting the “survivors” from the analysis, introduces a systematic bias, leading to an underestimation of lifetime.^15^

*Left-truncation*, or *delayed entry*, presents a distinct challenge, occurring when buildings are observed only after a certain time since construction (truncation time). In such cases, the analysis is conditional on the buildings having survived until the observation period. If the construction date for all buildings is known, left-truncation can be addressed by adjusting the risk set, i.e., marking delayed entry using the Kaplan–Meier estimator.^15^ If uncorrected, the resulting systematic bias leads to an overestimation of survival.^15^ For cohorts where the share of buildings demolished prior to the entry time is unknown, this bias cannot be corrected, and the survival function remains anchored to one at the time of entry.

Another common form of incomplete observation is *interval-censoring*, which occurs when the survival time is known to lie between two values,^9^ for example when a construction or demolition event is assigned to a time interval rather than a specific date. In building lifetime estimation, this form of censoring typically arises in studies where the data is reported at fixed intervals, e.g., periodic censuses or successive editions of maps. A common but imprecise approach is to treat the event as occurring at a fixed point within the interval: assuming its right end point overestimates survival, while assuming its midpoint underestimates parameter uncertainty.^21^ Correctly handling interval-censoring requires more elaborate survival analysis techniques such as the Turnbull estimator.^21^

### 2.2 Search Methods and Inclusion Criteria

Publications were selected through a database search (Web of Science and Scopus) covering publications from all years until 2025 (search performed in February 2026), followed by forward and backward reference snowballing. The query was (“building life span” OR “building lifespan” OR “building lifetime” OR “building service life”) AND (“distribution” OR “survival” OR “demolished”). We only consider publications that estimate building lifetime based on empirical data (i.e., using records of construction, demolition, stocks, or a combination of these). We exclude: 1) expert estimates and anecdotal evidence, 2) theoretical estimates of how long a building *is supposed* to last (such as building standards or predictive engineering models), 3) case studies of single buildings. All building typologies (residential, nonresidential) were considered. No geographic restrictions were applied; however, the review was limited to publications written in English. We included both peer-reviewed journal publications and gray literature (e.g., conference articles, technical reports). Details on the literature search process (e.g., search query choice, detailed exclusion criteria) can be found in the Supporting Information SI1.

### 2.3 Taxonomy of Estimation Approaches

As so often in research, data is the crux of deriving lifetime estimates. Ideally, one would know the full history of a building stock differentiated by cohort, including the systems’ inflows (constructions) and outflows (demolitions) over a time horizon of decades or centuries. The information available in practice almost always falls short of this, and the amount and type of data are key factors in the choice of methodology.

Using data type as the basis, we developed a taxonomy of lifetime estimation approaches, centered on methodological aspects that can introduce bias and on the approaches’ ability to correct it. The focus is on right-censoring, the most widespread censoring type in building lifetime data.

### 2.4 Lifetime Estimate Extraction and Harmonization

From each identified publication, one or more building lifetime estimates were extracted. The estimates were in various forms depending on the estimation methodology used and the level of detail of the displayed results. In the order of decreasing level of detail, the estimates can be in the form of nonparametric distribution, parametric distribution (e.g., Weibull or log-normal), or point estimate (median or mean). If multiple estimate types were available for the same building stock, a higher level of detail was preferred.

After extracting the lifetime estimates, they were harmonized to allow for comparison of median values. For parametric distributions and point estimates available as median, the extraction was straightforward. For nonparametric distributions, the medians were extracted whenever possible, i.e., for curves where stock survival reaches below 50%. The median was assumed to be the first available discrete point equal to or lower than 50%; the sensitivity of the model results were tested by assuming instead the last discrete point before reaching 50%. For point estimates available only as a mean μ, medians *M* were obtained by assuming a log-normal parametric distribution:

$$M=\mu /e^{\ln\left(CV^{2}+1\right)/2}$$

where *CV* is the coefficient of variation defined as the ratio of the standard deviation σ and the mean μ. We choose *CV* = 0.8 as proxy for converting mean values to medians based on the average of the log-normal parametric estimates collected in our sample.^11,22–24^ Values of 0.5 and 1.0 were used to test the sensitivity of the model results to *CV* assumptions.

### 2.5 Statistical Analysis

The statistical analysis was divided into two parts. First, the extracted median values were analyzed descriptively by observing the trends visible in the collected data without looking at covariate interdependencies. Second, statistical inference was applied to analyze the log-transformed median values using a mixed-effects meta regression model, which considers clustering of observations employing the same data source (details on clustering can be found in the Supporting Information SI1). Due to the absence of reported standard errors in most studies, the weight *w*_*i*_ for each estimate *i* was calculated using proxy for variance:

$$w_{i}=1/variance\approx n_{i}$$

where *n*_*i*_ is the sample size of estimate *i*. For estimates based on empirical demolition data, the sample size *n* represents the number of demolished buildings, while for estimates based on model calibration (Section 3.2), it represents the degrees of freedom (the number of independent calibration data points minus the number of fitted parameters). For model selection, we prioritized candidates that minimized the Akaike Information Criterion (AIC) while maintaining low Variance Inflation Factor (VIF) to maintain the stability and interpretability of the regression. The explanatory power of the final model was quantified using the coefficient of determination (*R* ^2^). The analysis was performed in R using the *Metafor* package. The final model followed the regression equation:

$$\log\left(t_{50,i}\right)=\beta_{0}+\overset{F}{\underset{f=1}{\sum }}\overset{L_{f}}{\underset{l=2}{\sum }}\beta_{f,l}\cdot l\left[X_{f,i}=l\right]+u_{m\left(i\right)}+\varepsilon_{i}$$

where *t*_50,_ *_i_* is the median lifetime value for observation *i*; β_0_ is the intercept, representing the baseline when all factors are at their reference level; β_*f*,*l*_ is the fixed-effect coefficient for level *l* of factor *f*, *f* is the number of factors retained in the final model, *L*_*f*_ is the number of levels of factor *f*, with level 1 as reference; l[] is the indicator function (1 if true, 0 otherwise); *X*_*f*,*i*_ is the level of factor *f* for observation *i*, *u*_*m*(*i*)_ is the random effect for grouping unit *m* (each data cluster) assigned to each observation *i*, ε_*i*_ is the residual effect for observation *i*. A list of factors and corresponding levels can be found in Table 1. Left-truncation was left out of the analysis due to high collinearity with other predictors (VIF > 10). The code is available at https://github.com/ecological-systems-design/building-lifetimes-lit-review. The collected median lifetimes as well as regression-predicted values are listed in Supporting Information SI2.

**Table 1:** Coding of the Covariates Used in the Final Model^a^

Factor *f*		Level *l* of factor *f*	
No.	Name	No.	Name
1	Use class	1	Residential*
		2	All
		3	Nonresidential
2	World region	1	Asia*
		2	Europe
		3	North America and New Zealand
3	Spatial coverage	1	National/subnational*
		2	City/neighborhood
4	Cohort group	1	Post-1950*
		2	Unspecified
		3	Pre-1950
5	Structural material	1	Nonwooden*
		2	Unspecified
		3	Wooden
6	Right censoring	1	Corrected/not applicable*
		2	Not corrected

a Asterisk (*) denotes the reference category (level 1 for each factor).

## 3 Results

### 3.1 Overview of Studies

We identified 46 relevant sources, including journal articles (38), conference articles (5), technical reports (2) and a book chapter (1). The journal publications were predominantly in the fields of building engineering, architecture, sustainability, and industrial ecology. The studies were published between 1985 and 2025 (Figure 1). In the majority of cases, building lifetimes were the main purpose of the study (27), but in some cases lifetime estimation was a means to investigate building stock dynamics (16), or environmental impact assessment (3). Most publications (30 of 46) were found through reference snowballing, reflecting the difficulty of choosing a precise yet sensitive query due to terminological fragmentation (see Supporting Information SI1).

**Figure 1:**
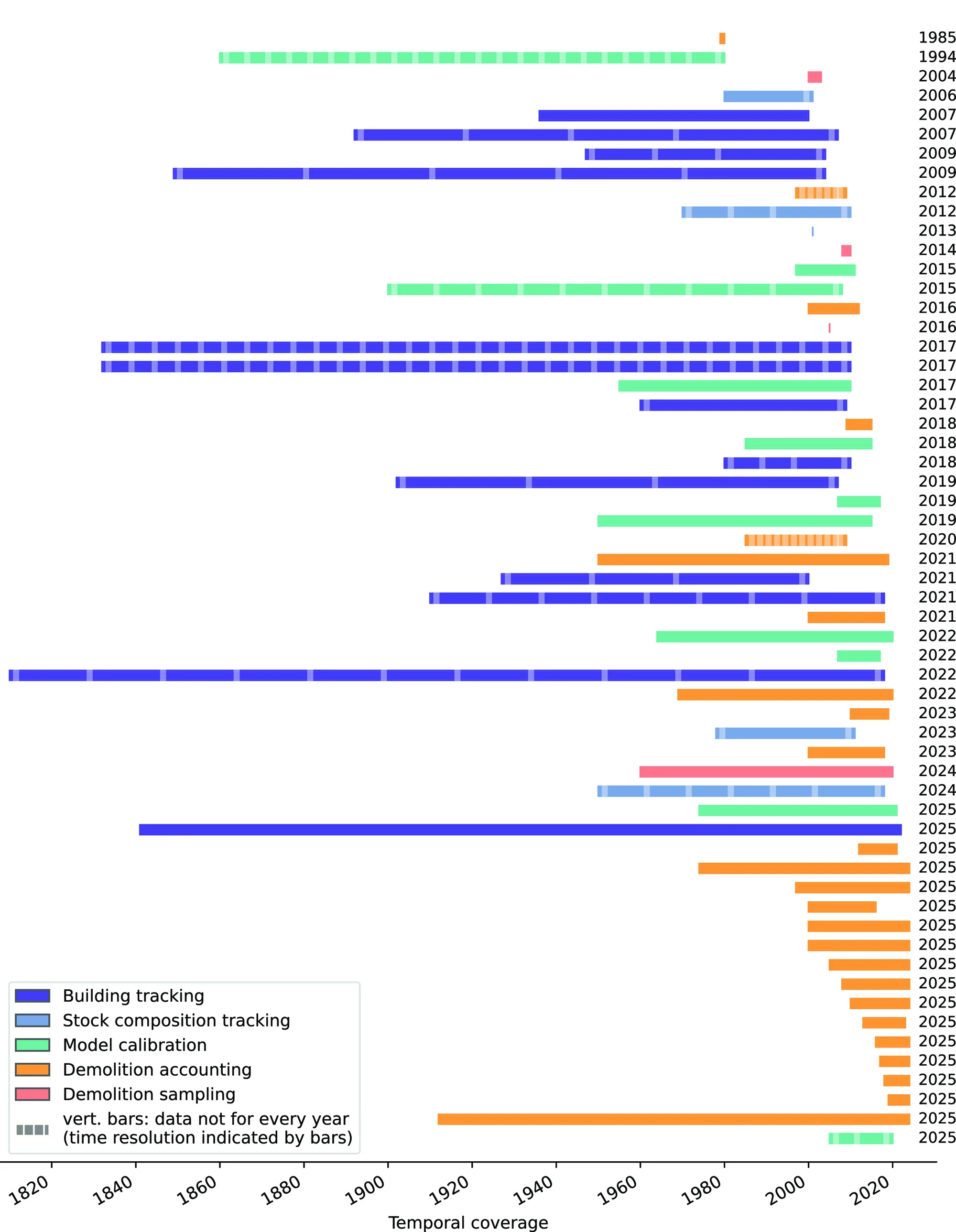
All case studies (59) by year of publication (on the right), the temporal coverage (extend of the bar on the *x*-axis), the temporal data resolution (distance between vertical bars equals average time between data points—no bars means separate data for each covered year), and the method used (color). For model calibration studies, the temporal coverage reflects the model inflow data.

Within the identified studies, some investigated more than one building stock. Berglund-Brown et al.^25^ performed 13 case studies for different cities scattered across Europe and North America. Tanikawa and Hashimoto^26^ studied building stocks in Japan and the UK. Altogether, we identified 59 case studies on building stock lifetime estimation.

### 3.2 Lifetime Estimation Approaches

We identified five lifetime estimation approaches (Table 2) distinguished primarily by empirical data type, which determines the aggregation level and the ability to consider right-censoring. Below we present each approach and exemplify it with a typical case illustrating the data used and the lifetime estimate obtained (Figure 2).

**Table 2:** Overview of the Lifetime Estimation Approaches Used in the Identified Publications

No.	Approach name	Typical data type	Aggregation level	Can account for right-censoring (the buildings still standing)?	Number of publications
1	Model calibration	Construction and aggregated stock statistics	Stock	Not applicable (stock represented via mathematical modeling)	12 ^22–24,27–35^
2	Stock composition tracking	Census data with stock composition by cohort	Stock	Yes	5 ^36–40^
3	Building tracking	Historical maps and aerial photos	Building unit	Yes	12 ^3,11,14,26,41–48^
4	Demolition sampling	Demolition survey	Building unit	No	4 ^49–52^
5	Demolition accounting	Building cadaster	Building unit	Usually yes (if stock data is available)	13 ^2,5,25,53–61^
					Total: 46

**Figure 2:**
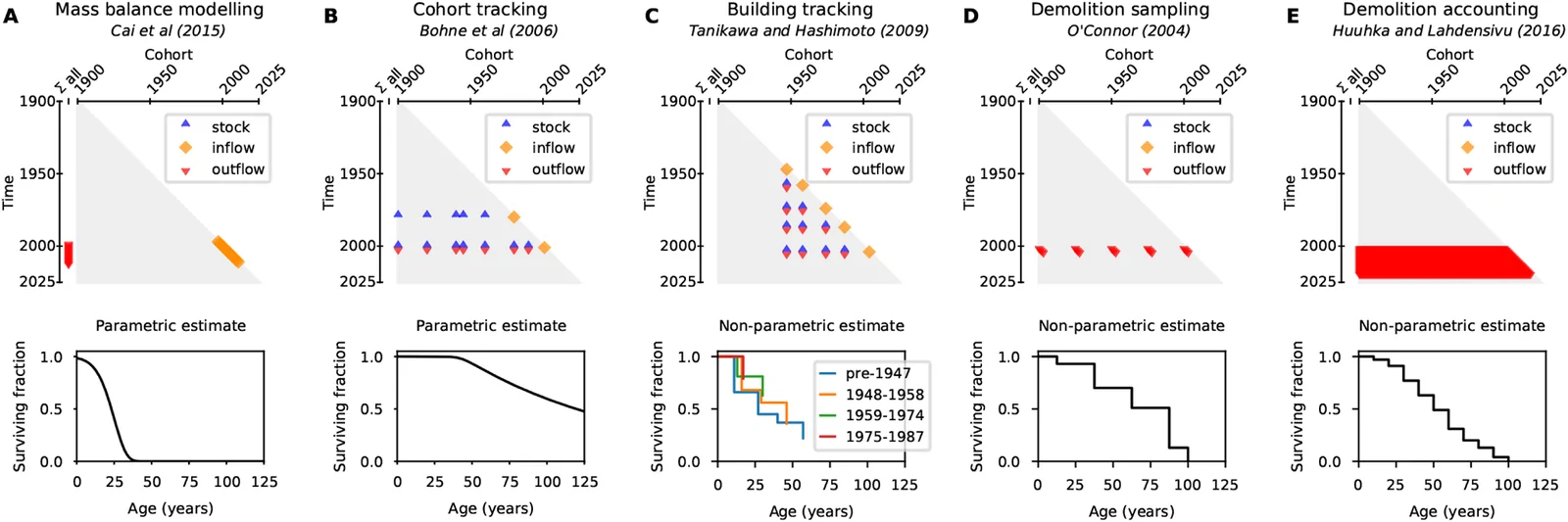
Examples of data used and results generated for each methodology. In each panel, the top plot displays the full data space in the time-cohort matrix (gray triangle) with the available study data (blue/red/orange markers). “∑ all” denotes the column with cohortless data. The bottom plot shows the survival curve estimates derived from this data. Note: the parametric estimate in panel B is truncated to align with the range of empirical data shown in the top plot; extrapolations beyond this limit lack empirical grounding and are therefore not shown.

#### 3.2.1 Approach 1: Model Calibration

Instead of direct empirical observation, this method utilizes the combination of inflow, stock and/or outflow data to mathematically reconstruct lifetime functions. A model is built, typically using historical construction series as input (input-driven model^8^), and the lifetime function is calibrated against aggregated stock data,^22–24,27–29,34,35^ aggregated demolition data,^31,32^ or similar.^30^ The use of aggregated data is typical of this approach (Figure 2A). This approach constitutes an inverse problem with multiple potential solutions, so researchers rely on parametric assumptions (e.g., Weibull or log-normal distributions) to stabilize the model. Calibration employs different optimization algorithms such as the least-squares method^22–24,30,32^ or Bayesian inference.^28^ The approach permits time-variant parametrization to fit distinct lifetime functions by cohort^22–24,27^ or period,^22,27^ although this necessitates additional assumptions, increasing the risk of overfitting. While these estimates are not formally impacted by censoring, they remain parametric approximations with a limited granularity. For example, as they are typically calibrated to recent demolition or stock data, the estimated mortality of the oldest buildings relies on the extrapolation of the fitted distribution rather than empirical evidence of late-stage survival (see the time scales in Figure 2A). In addition, some models simulate floorspace rather than individual buildings due to the units of the underlying model input and calibration data (e.g., refs 23,30 ).

#### 3.2.2 Approach 2: Stock Composition Tracking

This approach uses periodic building census data in which the stock is disaggregated by construction cohort groups. By comparing subsequent census snapshots of the stock, the attrition of a specific cohort can be tracked over time, with demolitions inferred from the net decrease in cohort volume. While tracking over multiple decades allows for a longitudinal reconstruction of survival curves for specific cohorts,^37^ two snapshots across multiple cohort groups are sufficient to construct a cross-sectional “synthetic cohort” ^36,39,40^ (a horizontal line in the time-cohort matrix in Figure 2B). One study estimated lifetime from a single census snapshot^38^ by assuming that buildings marked in the census as “in ruinous state” will soon be demolished. Interval-censoring may be a concern since the underlying census data is published periodically, for example every 10 years.^40^ Right-censoring affects estimates obtained using this approach but can be accounted for as the stock level is known. While this and the following approaches are based on data allowing for nonparametric lifetime estimates, sometimes only parametric approximations or point estimates are given (see Figure 2B).

#### 3.2.3 Approach 3: Building Tracking

In this method, a high-resolution data set is constructed by tracing individual buildings via addresses, building identifiers, or geographical coordinates across diverse sources, including cadasters, historical maps, aerial photographs, city archives, and insurance records. Each building is followed longitudinally from the moment it first appears in the records until its disappearance (vertical lines in the time-cohort matrix in Figure 2C). While labor-intensive, this method offers unparalleled period coverage; some observation periods extend over 150 years.^26,41–43^ However, data requirements typically restrict the scope to city- or neighborhood-level studies in data-rich contexts like Switzerland,^41,43^ Germany,^14^ Japan,^3,26^ Norway,^42^ or the United States.^11^ The collected data sets sometimes include individual building metadata such as use class^3,14,26,41,45,48^ or volume.^41^ Left-truncation arises when the construction of longitudinally analyzed cohorts precedes the observation period. While the impact of left-truncation often remains opaque in the literature, the oldest cohorts in Bradley and Kohler^14^ clearly illustrate the bias, where left-truncation causes unrealistically high survival rates in older cohorts. Right-censoring is implicitly accounted for, as can be observed for example through truncated survival curves, representing the share of the surviving stock (Figure 2C). Interval-censoring is common, as stock snapshots may be separated by decades, leaving the exact timing of demolition events between snapshots unknown (e.g., ref 26). While building tracking can in principle achieve full coverage of a studied area, record completeness in practice depends on the quality of underlying sources (e.g., map resolution) and the accuracy of extraction methods.

#### 3.2.4 Approach 4: Demolition Sampling

Studies using this approach rely on demolition data obtained through sampling, resulting in a cross-sectional estimate (Figure 2D). These surveys collect construction and demolition dates for a subset of buildings, sometimes alongside individual building metadata such as building use class,^49,50^ structural material,^50^ dimensions^49^ or reason for demolition.^50^ The sample size is typically small, with some surveys reporting as few as 38 buildings.^52^ Although the data is typically collected over several years, demolition records are usually combined into a single data set, so changes over time are not investigated. Right-censoring bias is inherent to this method but cannot be corrected due to the lack of information about the entire building population and thus the remaining stock of each cohort. Sampling may also not be representative of the entire population, introducing additional bias. Some surveys ask about the building age indicated as a range, resulting in interval censoring (Figure 2D). The representativeness of the resulting estimates depends on the sample size and sampling technique.

#### 3.2.5 Approach 5: Demolition Accounting

This approach uses routinely collected demolition data, which report the cohort year of each demolished building in a given stock (Figure 2E). The data is typically obtained from administrative public records such as national building cadasters,^5,54–56^ but may also come from private records of the building industry^62^ or public information.^61^ The records may include individual building metadata, such as use class,^25,54–56^ structural material,^5,25^ roof type,^61^ refurbishment state,^56^ or reason for demolition.^55^ The data from multiple years is typically pooled into a single data set, preventing an analysis of lifetime change by period (with one notable exception^54^). Left-truncation and right-censoring are inherent to this method and bias the results unless accounted for. Some studies consider right-censoring,^53,61,62^ but most estimates are uncorrected. This is despite, in many cases, the data comes from sources where the remaining stock at the end of the study is likely known (e.g., refs 2, 5, 54–57,60 ). Period coverage typically begins in the 2000s–2010s, coinciding with the start of formal record-keeping. Exceptions include studies using city-level data,^25^ those with incomplete historical records,^5^ or those focusing on private records^62^ or specific subsets like public sports stadiums.^61^ Data completeness depends on whether the underlying data set represents the full building stock of a given area or only a stock subset (e.g., a stock sample from a survey^2,53^ or records limited to a single construction company,^62^ in which case representativeness of that subset also becomes relevant). Completeness additionally depends on whether construction year is consistently recorded for all demolished buildings.

### 3.3 Overview of Lifetime Estimates

In total, 336 lifetime estimates were extracted from 59 case studies identified within 46 publications. Some case studies involved more than one estimate. For example, Tanikawa and Hashimoto^26^ and Guo et al.^46^ listed several estimates varying by factors such as building use class, subclass and construction cohort, and Daigo et al.^22^ included estimates by structural material and estimation period. The extracted estimates were of various types: nonparametric (208), parametric (61) and point estimates (67). Point estimates were predominantly expressed as mean values (49), followed by medians (18). Detailed results of the classification can be found in Supporting Information SI2.

Out of 208 nonparametric estimates, 78 were truncated before reaching the survival rate of 0.5 and therefore were excluded from the statistical analysis of medians. Due to this exclusion, four^37,39,45,62^ out of the identified 46 studies were not represented in the final lifetime estimate sample. Collecting all the extracted medians, including those obtained by applying proxy to mean point estimates (see Section 2.4), resulted in 258 median building lifetime estimates.

The obtained estimates followed patterns partly dictated by the estimation method (Figure 3). The model calibration approach naturally yielded parametric estimates (with one exception using Bayesian inference^28^). Building tracking and demolition sampling, as rather data-intensive approaches, were typically limited to city or neighborhood spatial coverage. We also noted a correlation between the method and world region, with model calibration used predominantly in Asia, and building tracking and stock composition tracking approaches used predominantly in Europe.

**Figure 3:**
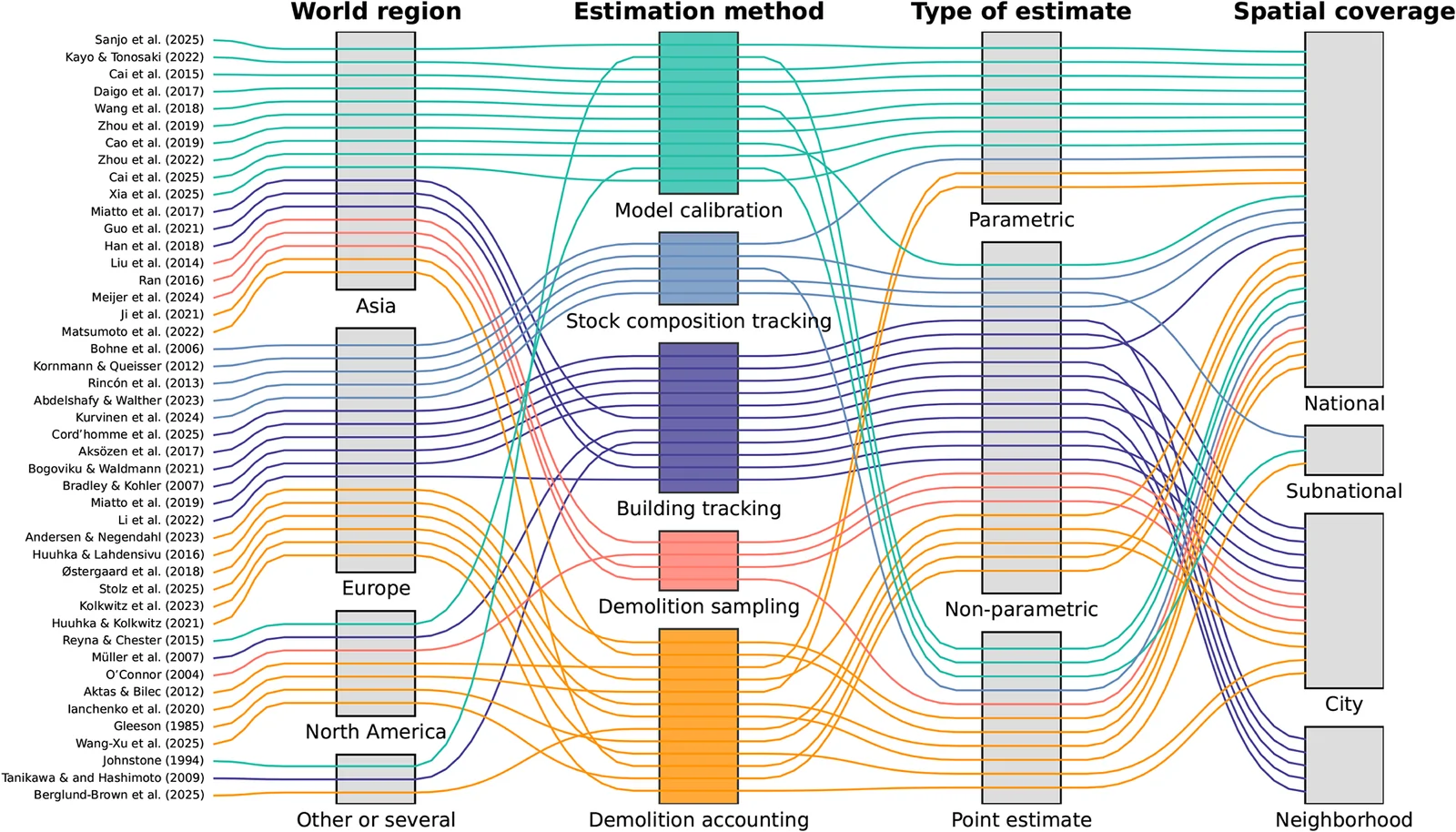
Alluvial chart connecting references with the world region they represent, type of estimation method, predominant type of retrievable lifetime estimate, and spatial coverage. The world region “Other” includes one study from New Zealand and two studies covering more than one world region.

### 3.4 Descriptive Statistics

In total, we extracted 258 median values from 336 identified lifetime estimates. These values are subject to various types of bias and therefore reflect the collected evidence rather than the global building stock itself. Note that these descriptive results are affected by confounding due to factor covariance, which is partially accounted for in the regression analysis in Section 3.5. The numeric results presented in the following two sections should not be used directly as modeling inputs without accounting for biases, uncertainties, and sensitivities (see Section 4.3 and Section 4.4).

The medians ranged from 6 to 323 years, with 50% of the central observations lying between 31 and 79 years (25th and 75th percentile). The median of medians was 46 years, and the mean of medians was 60 years, indicating a right-skewed distribution. The estimates varied across factors such as use class, world region, spatial coverage, and cohort group (Figure 4). The medians grouped by estimation approach and estimate type can be found in the Supporting Information SI1.

**Figure 4:**
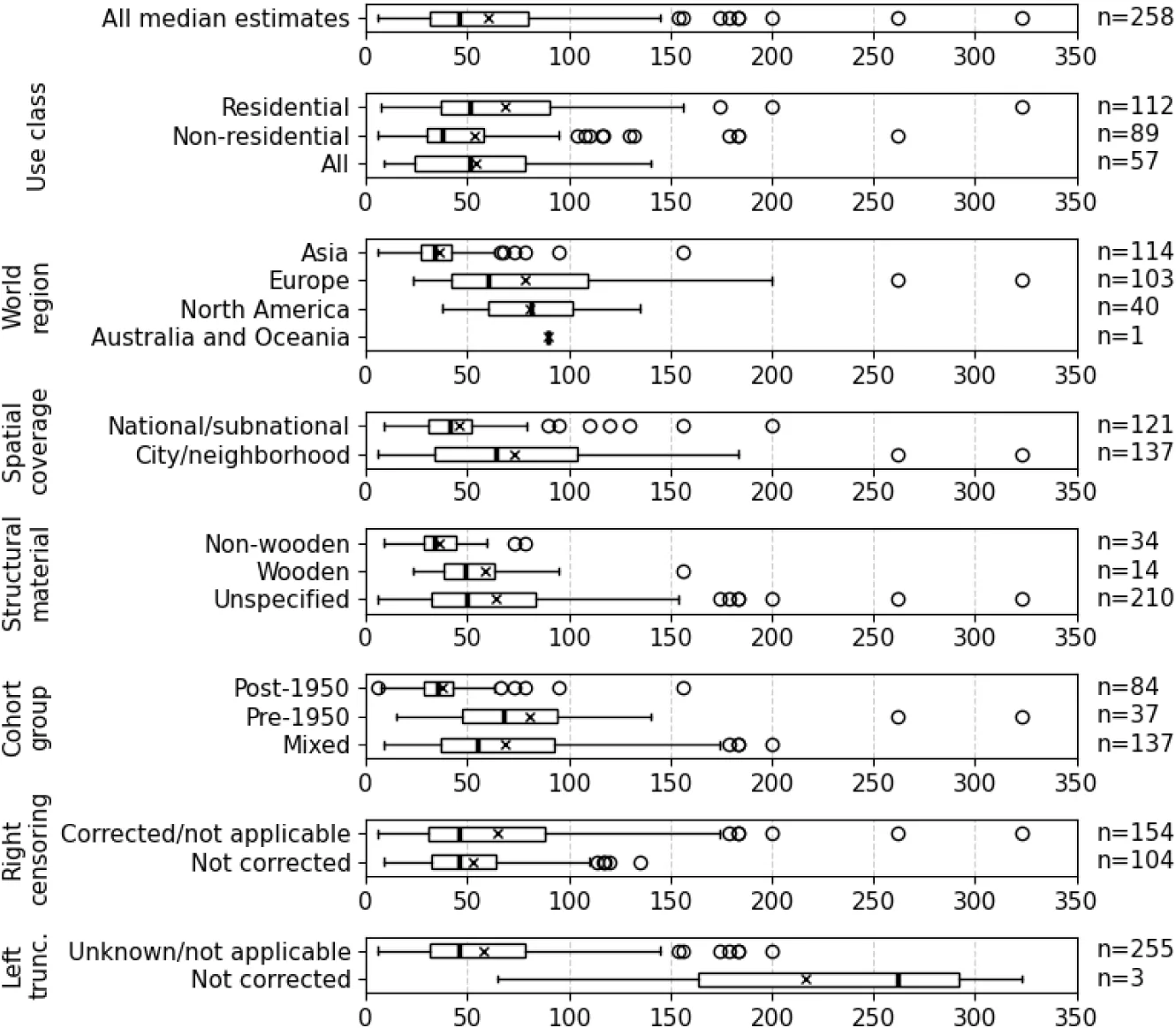
Statistical description of reported median estimates. The horizontal rectangle denotes the 25th–75th percentile range, the vertical thick line denotes the median, the cross denotes the mean. Note that these descriptive values are affected by confounding due to factor covariance, which is partially accounted for in the regression analysis in Section 3.5.

#### 3.4.1 Use Class

Across the included case studies, residential stocks consistently exhibited higher median lifetimes (51 years) than nonresidential stocks (37 years; −26%). Individual studies reported values of −60% to 11% in Europe^14,26,41,50,54–56^ and −12% to 4% in Asia.^22,26,49^ While no studies have explicitly examined mixed-use buildings, indications suggest that those with a predominance of nonresidential use have shorter building lifetimes compared to those where residential use predominates.^42^

On a use subclass level, a more comprehensive analysis was limited due to few data points and inconsistent naming convention. For agricultural buildings, we observed a high variance even within similar regional contexts (Denmark and Finland^54–56^ ). In several European countries, lower-density housing such as detached houses lasted longer than multifamily housing.^26,54,55^ However, the opposite trend was reported in Japan, with high-density housing exhibiting longer lifetimes than lower-density typologies.^26^

#### 3.4.2 World Region and Spatial Context

Among the reviewed studies, we observed a regional divide: building longevity reported in Asia (median 34 years) was much lower than in Europe (60 years; +78%) and North America (81 years; +141%). While few studies investigated subnational differences, hindering a statistical analysis, national averages were observed to be higher than estimates for metropolitan areas in Spain,^38^ Denmark^56^ and Japan.^24^

In contrast, the identified national-level estimates (median 41 years) were lower than city- and neighborhood-level studies (64 years; +56%). The highest values were reported in central historical areas, such as in the core district of Ettlingen (Germany),^14^ the city center of Trondheim (Norway),^42^ central Zurich (Switzerland),^41,43^ Padua (Italy),^45^ and New Haven (United States).^11^ In Zurich, buildings in the historical city district had a median lifetime of 250 years compared to 110–140 years in other districts.^41^ Similarly, in Trondheim, the central neighborhood exhibited average lifetimes exceeding 110 years,^42^ whereas other neighborhoods within the same city averaged only 35–45 years.^57^

#### 3.4.3 Physical Attributes

In the literature captured by this review, buildings with nonwooden structural material such as reinforced concrete exhibited shorter lifetimes (34 years) than wooden ones (49 years; +44%). Two individual studies reported that wooden structures are associated with 50–60% longer lifetimes compared to nonwooden counterparts.^5,24^ Regarding nonstructural materials, a study in Denmark found that heavy wall materials (timber and brick) were linked to longer lifetimes compared to light materials (wooden paneling and sheet metal).^56^

Other building attributes could not be subject to statistical analysis due to few data points. Individual studies suggested that buildings of larger sizes survive longer. In Zurich,^41^ longer lifetimes were reported for buildings with medium-to-high volume and medium footprint. Similarly, in Chongqing (China),^49^ lower demolition risks were reported for buildings with larger floor areas. Building condition and renovation status also played a role; for example, in Denmark, refurbished buildings were shown to live on average 7% longer than nonrefurbished ones.^56^

#### 3.4.4 Temporal Dynamics

The available evidence showed that post-1950 cohorts exhibited shorter reported lifetimes (median 35 years) than pre-1950 ones (68 years; +94%). A similar trend of decreasing lifetime by construction cohort was observed in several individual studies,^14,23,30,43,54^ although others found no clear correlation between construction year and demolition rate.^22,24,44,45^

Cross-sectional studies with period lifetime measurement were few and with large temporal overlap, hindering a statistical analysis of how lifetimes changed cross-sectionally over time. However, individual studies observed period trends in lifetimes and demolition patterns. For example, Bradley and Kohler^14^ observed demolition peaks in the mid-1950s and the 1980s, and Aksözen et al.^43^ observed a steady decrease of age at demolition over time.^43^ Ianchenko et al.^53^ observed a decrease in demolition rates in 2007–2009 and linked it to economic conditions and lower construction activity in the US. Finally, Cord’homme et al.^42^ analyzed building mortality over time, linking it with specific shocks such as fires, wars, and the introduction of heritage preservation acts.

#### 3.4.5 Lifetime Estimation Bias

Due to confounding (see regression analysis, Section 3.5), the descriptive evidence showed no visible impact of right-censoring on reported median lifetimes, with uncorrected studies showing the same value as corrected ones (median 46 years). In contrast, left-truncation issues produced visible outliers (median 262 years), although these cases remained infrequent compared with studies in which the effect was absent or undocumented (46 years).

### 3.5 Inferential Statistics

To isolate the independent effect of each factor, we performed a mixed-effects meta-regression analysis of log-transformed median building lifetimes. This inferential analysis corrects for confounding among factors documented in the collected evidence.

The model (Table 3) explained a substantial portion of the heterogeneity (*R* ^2^ = 0.85). The normality assumption of the model was met (see Figure S2 in Supporting Information SI1). The reference profile represented a residential, nonwooden building from a post-1950 cohort in Asia, as reported by national- or subnational-level studies where right-censoring was corrected or not applicable. The median lifetime for this reference was calculated at 23.36 years (95% CI: 17.31–31.53, *p* < 0.001). The median lifetimes for all factor combinations and predicted median lifetimes for individual observations can be found in Supporting Information SI2.

**Table 3:** Cluster-Robust Coefficients for the Meta-Regression Model^a^

Factor	Level	Estimate	SE	*p*-value	95% CI	Exp (Estimate) [95% CI]
Intercept	-	3.15	0.15	<0.001***	[2.85,3.45]	23.36 [17.31,31.53]
Use class (Ref: residential)	All	–0.29	0.14	0.05	[−0.59,0.00]	0.74 [0.55,1.00]
	Nonresidential	–0.26	0.04	<0.001***	[−0.33,–0.18]	0.77 [0.72,0.83]
World region (Ref: Asia)	Europe	0.90	0.16	<0.001***	[0.58,1.23]	2.46 [1.78,3.41]
	North America and New Zealand	0.99	0.15	<0.001***	[0.70,1.29]	2.70 [2.00,3.63]
Spatial coverage (Ref: National/subnational)	City/neighborhood	0.29	0.13	<0.05*	[0.03,0.56]	1.34 [1.03,1.75]
Cohort group (Ref: Post-1950)	Mixed	0.56	0.00	<0.001***	[0.56,0.56]	1.75 [1.75,1.75]
	Pre-1950	0.19	0.12	0.108	[−0.04,0.43]	1.21 [0.96,1.54]
Structural material group (Ref: Nonwooden)	Unspecified	0.16	0.00	<0.001***	[0.16,0.16]	1.17 [1.17,1.17]
	Wooden	0.83	0.00	<0.001***		2.30 [2.30,2.31]
Right-censoring (Ref: Corrected/not applicable)	Not corrected	–0.69	0.01	<0.001***	[−0.70,–0.68]	0.50 [0.49,0.51]

a “Ref” denotes the reference category for each factor. Estimates, standard errors (SE), and 95% confidence intervals (CI) are reported on the log-scale. For the intercept, “Exp (Estimate)” represents the predicted median lifetime of the reference profile; for all other factors, it represents the Ratio of Medians (RoM). Asterisks indicate statistical significance levels: * *p* < 0.05, ** *p* < 0.01, and *** *p* < 0.001.

Use class and world region stood out in the model as primary predictors of longevity. Nonresidential buildings exhibited a lifetime only 77% as long as the residential reference (Ratio of Medians [RoM] = 0.77, 95% CI: 0.72–0.83, *p* < 0.01), corresponding to an estimated 18.06 years (95% CI: 13.11–24.88). Compared to Asia, median lifetimes were estimated to be 2.46 times longer in Europe (95% CI: 1.78–3.41, *p* < 0.001), and 2.70 times longer in North America and New Zealand (95% CI: 2.00–3.63, *p* < 0.001).

Structural material also appeared as a significant predictor: reported wooden buildings demonstrated higher longevity, with a multiplicative effect of 2.30 (95% CI: 2.30–2.31, *p* < 0.001) compared to nonwooden structures. However, the near-zero standard error represents an identifiability issue between structural material and world region since wooden buildings appeared almost exclusively in Asia. Additionally, mixed construction cohorts appeared to have longer lifetimes compared to post-1950 cohorts (RoM = 1.75, 95% CI: 1.75–1.75, *p* < 0.001), and studies with spatial coverage at the city/neighborhood exhibited longer lifetimes than national/subnational scope (RoM = 1.34, 95% CI: 1.03–1.75).

The model revealed a major methodological bias: studies failing to correct for right-censoring reported lifetimes approximately 50% as long as those accounting for censoring (RoM = 0.50, 95% CI: 0.49–0.51, *p* < 0.001). In fact, our sample contains two studies that are identical in their choice of data source and method but different in the bias treatment, and the difference in their median lifetime is almost exactly 50%.^2,53^ In contrast, spatial coverage construction cohort (pre- vs post-1950) was not a significant predictor (*p* > 0.05) when other covariates were held constant. This suggests that the trend observed in the descriptive analysis was likely confounded by other covariates, for example, the right-censoring bias might have affected the perceived difference between pre- and post-1950 cohorts.

To assess robustness, we tested sensitivity to two assumptions: the coefficient of variation used to convert means to medians, and the method for extracting medians from nonparametric estimates. The former did not affect factor significance, while the latter affected the spatial coverage factor (*p* = 0.032), which was significant but without a wide margin. All other covariates, including nonresidential use class, world region, structural material, and right censoring, remained significant across all specifications; in addition, the right-censoring bias estimation remained unchanged.

## 4 Discussion

### 4.1 Current Empirical Evidence and Data Gaps

We reviewed 46 studies and analyzed 258 median lifetime estimates, demonstrating that building stocks exhibit significant lifetime heterogeneity across world regions, use classes, and structural materials. While the lack of large-scale global studies hinders a direct cross-regional comparison, the observed range of lifetimes in Asian stocks is consistent with the findings of Murakami et al.^6^

The review highlights several critical data gaps. Geographically, the evidence base lacks representation from high-growth nations (e.g., India, Indonesia) and entire regions, including South America and Africa. While national- and city-level analyses are common, rural and suburban contexts are rarely addressed. Rare exceptions include metropolitan-scale studies^34,57^ and national studies that differentiate by region^5,33,38^ or by urban-rural split.^35^ Research rarely investigated specific use classes, such as the distinction between single-family and multifamily housing.^54,55,60^ Notably absent are studies on mixed-use buildings and informal housing, the latter being a widespread typology in many low- and middle-income countries.^63^ These data gaps represent a potential risk to the accuracy of global carbon budgeting, as high construction volumes and associated carbon footprint are expected in regions such as India, Latin America and the Middle East,^64^ which currently lack empirical evidence on stock turnover times. As shown in this review, these turnover times may differ across geographical contexts by a factor of 2 or more.

While our analysis focused on median values, the collected estimates reveal a systematic blind spot regarding mature stocks and “late survivors”. Only 11 of the 46 identified studies provide empirical data on survival rates beyond 25%, meaning that the current modeling of long-lived stocks relies heavily on the parametric extrapolation of earlier mortality patterns. Such poor empirical grounding for the “long tail” of building survival rates introduces uncertainty to long-term material flow modeling, potentially underestimating carbon lock-in effects, as older stocks are often under heritage protection.^42^ Furthermore, our limited understanding of these “late survivors” restricts policymakers’ ability to design evidence-based lifetime extension strategies. Without understanding how longevity correlates with specific drivers, such as design factors, zoning regulations, and heritage protection laws, we risk prioritizing new construction over retaining the value of the existing building stock.

### 4.2 Methodology for Lifetime Estimation

While previous work provided similar taxonomies,^11,12^ our classification is centered on censoring and its mitigation using survival analysis tools. For example, we differentiate between demolition sampling (Approach 4) and accounting (Approach 5), a distinction overlooked in previous work yet critical for the formal consideration of right-censoring. Consequently, this classification provides a robust framework for future empirical research, guiding researchers in matching available data types to appropriate estimation methods while identifying the methodological biases and the potential for correction.

Our review reveals significant bias in existing estimates. For example, some reported medians exceed the observation period, such as the oldest cohorts in Bradley and Kohler^14^ exceeding 250 years, overestimated due to left-truncation. Similarly, some estimates for post-1950 cohorts exceed 75 years,^11,24^ a value that is empirically impossible to observe in 2025 and is instead a byproduct of parametric extrapolation. Yet the most pervasive issue is uncorrected right-censoring bias, affecting 104 of the collected 258 median lifetimes, where only demolished buildings are considered, and buildings that remain standing are omitted from the analysis. While some authors acknowledge this issue by using terms like “age at demolition”^55^ or “lifetimes of demolished buildings”,^25^ practitioners not familiar with survival analysis may continue to use these figures in BSM, resulting in underestimated lifetime projections. Our meta-analysis quantified the severity of this issue, showing that uncorrected estimates are around 50% lower than those corrected for right-censoring or unaffected by it. We identified a large “lost opportunity” for accuracy: among 13 studies applying a demolition accounting approach, the majority likely had access to stock data required for right-censoring bias correction yet only three applied it.^53,61,62^ These findings underscore the need to integrate survival analysis concepts such as right-censoring into BSM. Failure to do so risks further proliferation of biased empirical estimates and the use of these in modeling, leading to skewed projections of resource use and emissions trajectories.

Finally, our methodological classification provides guidance in how data availability dictates the scope of achievable insights. For example, nearly all estimates for pre-1950 cohorts originate from labor-intensive longitudinal studies using building tracking, indicating that obtaining historical depth requires a significant resource investment in data reconstruction. Looking forward, this data collection bottleneck could be bypassed through automated pipelines leveraging remote sensing and machine learning, see for example Kleemann et al.^68^ Our framework also indicates that in data-scarce regions, where high-resolution longitudinal data is unavailable, national-level cross-sectional estimates remain feasible through aggregate-level approaches such as model calibration or stock composition tracking. Consequently, available data can be leveraged to maximize insight, while systematic blind spots can be addressed by targeted data collection and automation efforts.

### 4.3 Limitations

Interpretation of the presented evidence must account for several systematic biases. Due to the *coverage bias*, the collected evidence reflects what was being researched and published rather than the true target populations. This means that some regions were overrepresented due to data infrastructure, research funding, and publication practices. In addition, the decision to measure or report a specific predictor can depend on the local context, which is known as the *selective reporting bias*. For example, due to seismic safety concerns, structural material is more likely to be reported in Japan than in other countries.

The *selection bias* is the systematic exclusion of relevant literature caused by restricted search criteria. For example, our systematic search was limited to English-language publications, excluding some relevant empirical works (e.g., in Japanese^65,66^ and Dutch^67^). Notably, all pre-2006 studies in our sample originate from Anglophone countries. Furthermore, the identification of relevant studies may also have been affected by the lack of unified terminology across disciplines, with lifetimes variously referred to as, e.g., residence times or turnover rates.^34^ The terminological fragmentation meant that no search query we tested was both sensitive enough to capture most publications in our final sample (the remainder were identified via snowballing) and specific enough to yield a manageable number of records (see Supporting Information SI1).

The *imputation/extraction bias* reflects errors introduced when extracting and converting data for the analysis from methodologically heterogeneous source studies. Because primary data and standard errors were rarely reported, we relied on manual data extraction and used sample size as a proxy for variance. Reporting granularity additionally restricted our synthesis; in approximately one-third of the identified studies, it was impossible to retrieve data in its most descriptive format. For example, in many cases, parametric or nonparametric distributions were analyzed but only point estimates were reported in a readable format. In addition, due to the inherent differences between model calibration and other estimation methods, a distinct weighting approach was required for these estimates.

The *aggregation bias* is the distortion caused by pooling distinct subpopulations into one single category, resulting in uneven composition of the building stock within each category. For example, the world region “Asia” aggregates all Asian estimates irrespective of the true country-level composition, and the cohort group “pre-1950” pools all pre-1950 construction cohorts irrespective of the true cohort composition.

Finally, the *confounding bias* arises when systematic correlation occurs between covariates. The inferential analysis corrects for confounding among factors documented in the collected evidence; however, the cross-factor correlations are not always reported, preventing isolation of true effects. For example, while structural material was found to be a significant predictor, no data exists specifically for pre-1950 buildings, so the correlation between cohort and structural material cannot be verified.

Furthermore, our work considers only median lifetimes and does not quantitatively analyze the rest of the distribution, so no general conclusions can be drawn regarding shape, skewness, or suitable parametric functions, although most individual studies report log-normal or Weibull as the best-fitting function (see Supporting Information SI2).

### 4.4 Recommendations

Building on the findings of this study, we offer recommendations for two audiences: those using existing lifetime estimates, and those producing new ones. For users of existing lifetime estimates, we recommend the following:

- Be careful when extrapolating lifetime estimates for stocks beyond the scope of original studies, as this introduces uncertainty and bias (see Section 4.3).
- Be mindful of the potential right-censoring bias in the literature, as bias correction is not always clearly reported. For example, one right-censoring-corrected study in our sample only names the statistical software package used for survival analysis, without further explanation of the correction applied. Our classification can help with understanding where the risk is high (i.e., demolition sampling, demolition accounting).
- Consider preserving granularity when reusing lifetime study results: use the nonparametric estimate, or the full parametric distribution (e.g., log-normal, Weibull) as reported by the original authors, rather than creating and/or using single summary values such as the median.
- Check the sensitivity of your model results to lifetime assumptions, as building lifetimes are inherently heterogeneous and uncertain, particularly in prospective modeling.

For those producing new lifetime estimates, we recommend the following:

- Ensure transparency and reproducibility by providing the full raw data, allowing for the direct calculation of medians and standard errors; where data privacy constraints exist, provide a reproducible summary of data in the most descriptive format (usually nonparametric).
- Report lifetime estimates at the finest available granularity (e.g., use classes, construction cohorts, world regions), including joint combinations of factors (e.g., use class by structural material). This allows future syntheses to reaggregate data according to their own classification needs and to disentangle potential confounding among factors.
- Describe your methodological approach and define key terms explicitly (e.g., lifetime, use class types) particularly since shared conventions do not yet exist in the field.
- State clearly whether and how right-censoring applies to your methodological approach. If your method does not allow for correction (demolition sampling or demolition accounting without stock data), be transparent about the resulting underestimation of lifetimes. If your approach allows for correction (demolition accounting with stock data), use a method such as the Kaplan–Meier estimator.

## 5 Outlook

This study has provided a systematic review and meta-analysis of empirical building lifetime estimates, bridging building stock modeling research and survival analysis methodology. Looking ahead, several priorities emerge from our findings.

First, there is a pressing need to expand the evidence base. High-growth regions, such as India, Latin America and the Middle East, currently lack empirical data, yet are expected to experience high construction volumes and associated carbon footprints in the coming decades. Additionally, the lifetimes of informal housing and mixed-use typologies are poorly documented. Targeted studies in these contexts would substantially reduce uncertainty in global carbon budgeting. As shown in this review, even in data-scarce settings, building lifetime estimates may still be obtained using aggregate approaches or demolition sampling.

Second, our analysis reveals that uncorrected right-censoring bias is pervasive and leads to systematic underestimation of building lifetimes. Integrating survival analysis concepts into the standard practice of building stock modelers is essential to prevent the further proliferation of biased inputs into models. Increased transparency in reporting, ideally through the publication of raw data, would also enable more robust future synthesis.

Third, emerging technologies such as remote sensing and machine learning might in the near future provide researchers with tools to bypass current bottlenecks in empirical data collection and analysis. However, survival analysis literacy must keep pace with technological progress in the field, or else methodological complexity and black-box modeling may obscure the proliferation of systematic bias in the reported estimates.

Finally, while we showed a significant heterogeneity of building lifetimes across various predictors, the drivers of building demolition remained unaddressed. Future research should explore causal relationships to identify the underlying design, regulatory and socioeconomic factors responsible for fostering long-lived building stocks, and to support evidence-based policy for building lifetime extension.

## Supplementary Material





## Notes

S1: Systematic review and the statistical analysis (PDF)
S2: Classification of the identified publications, case studies, lifetime estimates, and median lifetime values (XLSX)
