# Supplementary material for: How Long Do Buildings Live: A Systematic Review and Meta-Analysis of Global Building Lifetimes
Source: Environ Sci Technol. 2026 Sep 8;60(37):26007–19. doi: 10.1021/acs.est.6c06705 (PMC13614209; doi:10.1021/acs.est.6c06705)
Supplement: Supplementary file 1 [file es6c06705_si_001.pdf]

Supporting information SI1

Title: How long do buildings live: a systematic review and meta-analysis of global building lifetimes

Authors: Kamila Krych[†], Nils Dittrich[‡], Daniel Müller[‡], Stefanie Hellweg[†]
[†] Institute of Environmental Engineering, ETH Zurich, Zurich, Switzerland
[‡] Department of Energy and Process Engineering, Norwegian University of Science and Technology, Trondheim, Norway

This supporting information provides details on the systematic review and the statistical analysis.

1 Systematic review

1.1 Details of the systematic review process

1. **Database search**. Using the selected query in a database search performed on 9 February 2026, we identified 65 items (46 items in Scopus and 19 items in Web of Science), out of which 18 were duplicates that we removed. One article was removed as the abstract could not be retrieved.
2. **Screening**. We screened 46 items based on title and abstract. Based on the screening, we excluded a total of 20 items due to various reasons:
   - No lifetime quantification (n=13), i.e., qualitative studies, quantitative studies but not engaging quantitatively with lifetime (e.g., “designed for disassembling and reassembling multiple times over the building's lifespan”);
   - Different definition of lifetime (n=7), i.e., quantitative studies on lifetime-related topics yet whose definition of lifetime is different from ours (likelihood of a demolition as a function of age t across the stock), e.g., reliability analysis in the context of seismic, load-bearing performance, or similar;
3. **Full-study assessment**. Out of the 26 items that were subjected to full-study assessment, we excluded a total of 10 items due to various reasons:
   - Cannot retrieve full paper (n=1);
   - Lifetime only as parameter (n=9), i.e., quantitative studies where lifetime is a model parameter whose value is assumed (no empirical investigation).
4. **Forward and backward reference snowballing**. The items included in the review (n=16) were all subjected to forward and backward reference snowballing. Backward snowballing was performed manually, by examining the reference list of every item. Forward snowballing was performed using search engines (Researchrabbit or Google Scholar), by examining all publications citing the chosen items. The identified publications were subjected to screening and full-study assessment procedure as shown in steps 2 and 3 above. Every publication identified through snowballing was subjected to snowballing itself until no new relevant publications were identified. As a result, 30 additional publications were identified.
5. **Final sample**. The final sample size was 46 items, including 16 identified through database search and 30 through reference snowballing.

1.2 Search query

The search query we used was: ("building life span" OR "building lifespan" OR "building lifetime" OR "building service life") AND ("distribution" OR "survival" OR "demolished"). This query is very specific, with around a third of items (excluding duplicates) relevant for our study. This query was chosen after testinf multiple other ones, which were more sensitive yet yielded too many records to be managaeble in a review.

For example, a more comprehensive query was tested: ("buildings" OR "building stock" OR "housing stock" OR "dwelling stock" OR "residential building") AND ("distribution" OR "survival" OR "demolished" OR "mortality" OR "lifetime" OR "lifespan" OR "life span" OR "service life" OR "longevity"). This query would manage to capture around 75% of the publications in our final sample yet yields 120,000 hits in Scopus.

A more specific query consisting of three keywords was also tested: ("buildings" OR "building stock" OR "housing stock" OR "dwelling stock" OR "material stock" OR "residential building") AND ("lifetime" OR "lifespan" OR "life span" OR "service life" OR "longevity") AND ("distribution" OR "survival" OR "demolished" OR "mortality"). This query would have been more managaeble in a review (close to 2,000 hits in Scopus); however, it would only allow to capture 5 extra publications from our final sample, still relying on snowballing for the recovery of most publications.

The difficulty choosing the right query and the reliance on snowballing for publication identification is ultimately the consequence of inconsistent terminology in building lifetime estimation research, which remains scattered across various scientific disciplines.

1.3 List of identified publications

Abdelshafy, A., & Walther, G. (2023). Using dynamic-locational material flow analysis to model the development of urban stock. Building Research & Information, 51(1), 5–20. https://doi.org/10.1080/09613218.2022.2142495

Aksözen, M., Hassler, U., & Kohler, N. (2017a). Reconstitution of the dynamics of an urban building stock. Building Research & Information, 45(3), 239–258. https://doi.org/10.1080/09613218.2016.1152040

Aksözen, M., Hassler, U., Rivallain, M., & Kohler, N. (2017b). Mortality analysis of an urban building stock. Building Research & Information, 45(3), 259–277. https://doi.org/10.1080/09613218.2016.1152531

Aktas, C. B., & Bilec, M. M. (2012). Impact of lifetime on US residential building LCA results. The International Journal of Life Cycle Assessment, 17(3), 337–349. https://doi.org/10.1007/s11367-011-0363-x

Andersen, R., & Negendahl, K. (2023). Lifespan prediction of existing building typologies. Journal of Building Engineering, 65, 105696. https://doi.org/10.1016/j.jobe.2022.105696

Berglund-Brown, J., Dobie, I., Hewitt, J., Wolf, C. D., & Ochsendorf, J. (2025). Lifetimes of demolished buildings in US and European cities. Buildings & Cities, 6(1). https://doi.org/10.5334/bc.588

Bogoviku, L., & Waldmann, D. (2021). Modelling of mineral construction and demolition waste dynamics through a combination of geospatial and image analysis. Journal of Environmental Management, 282, 111879. https://doi.org/10.1016/j.jenvman.2020.111879

Bohne, R. A., Brattebø, H., Bergsdal, H., & Hovde, P. J. (2006). Estimation of the service life of residential buildings, and building components. Norway. In Proceedings of the City Surface of Tomorrow Conference, Vienna, Austria, 8–9.

Bradley, P. E., & Kohler, N. (2007). Methodology for the survival analysis of urban building stocks. Building Research & Information, 35(5), 529–542. https://doi.org/10.1080/09613210701266939

Cai, W., Tian, P., You, K., Yu, Y., & Li, Y. (2025). Mitigating or Pushing? Unravelling the dual impact of lifetime on the CO2 Emissions from China's Urban Residential Building sector. Building and Environment, 281. Scopus. https://doi.org/10.1016/j.buildenv.2025.113236

Cai, W., Wan, L., Jiang, Y., Wang, C., & Lin, L. (2015). Short-Lived Buildings in China: Impacts on Water, Energy, and Carbon Emissions. Environmental Science & Technology, 49(24), Article 24. https://doi.org/10.1021/acs.est.5b02333

Cao, Z., Liu, G., Duan, H., Xi, F., Liu, G., & Yang, W. (2019). Unravelling the mystery of Chinese building lifetime: A calibration and verification based on dynamic material flow analysis. Applied Energy, 238, 442–452. https://doi.org/10.1016/j.apenergy.2019.01.106

Cord'homme, Z., Dittrich, N., Bringedal Gedde, K., Billy, R. G., & Müller, D. B. (2025). Approaching building lifetimes and hazard rates through demolition patterns: Case studies for a historical city center. Journal of Industrial Ecology, 29(1). https://doi.org/10.1111/jiec.13604

Daigo, I., Iwata, K., Oguchi, M., & Goto, Y. (2017). Lifetime Distribution of Buildings Decided by Economic Situation at Demolition: D-based Lifetime Distribution. Procedia CIRP, The 24th CIRP Conference on Life Cycle Engineering, 61, 146–151. https://doi.org/10.1016/j.procir.2016.11.221

Gleeson, M. E. (1985). Estimating Housing Mortality from Loss Records. Environment and Planning A: Economy and Space, 17(5), 647–659. https://doi.org/10.1068/a170647

Guo, J., Fishman, T., Wang, Y., Miatto, A., Wuyts, W., Zheng, L., Wang, H., & Tanikawa, H. (2021). Urban development and sustainability challenges chronicled by a century of construction material flows and stocks in Tiexi, China. Journal of Industrial Ecology, 25(1), 162–175. https://doi.org/10.1111/jiec.13054

Han, J., Chen, W.-Q., Zhang, L., & Liu, G. (2018). Uncovering the Spatiotemporal Dynamics of Urban Infrastructure Development: A High Spatial Resolution Material Stock and Flow Analysis. Environmental Science & Technology, 52(21), 12122–12132. https://doi.org/10.1021/acs.est.8b03111

Huuhka, S., & Kolkwitz, M. (2021). Stocks and flows of buildings: Analysis of existing, demolished, and constructed buildings in Tampere, Finland, 2000–2018. Journal of Industrial Ecology, 25(4), 948–960. https://doi.org/10.1111/jiec.13107

Huuhka, S., & Lahdensivu, J. (2016). Statistical and geographical study on demolished buildings. Building Research & Information, 44(1), 73–96. https://doi.org/10.1080/09613218.2014.980101

Ianchenko, A., Simonen, K., & Barnes, C. (2020). Residential Building Lifespan and Community Turnover. Journal of Architectural Engineering, 26(3), 04020026. https://doi.org/10.1061/(ASCE)AE.1943-5568.0000401

Ji, S., Lee, B., & Yi, M. Y. (2021). Building life-span prediction for life cycle assessment and life cycle cost using machine learning: A big data approach. Building and Environment, 205, 108267. https://doi.org/10.1016/j.buildenv.2021.108267

Johnstone, I. M. (1994). The Mortality of New Zealand Housing Stock. Architectural Science Review, 37(4), 181–188. https://doi.org/10.1080/00038628.1994.9697345

Kayo, C., & Tonosaki, M. (2022). Lifetimes of buildings in Japan. Resources, Conservation and Recycling, 185, 106504. https://doi.org/10.1016/j.resconrec.2022.106504

Kolkwitz, M., Luotonen, E., & Huuhka, S. (2023). How changes in urban morphology translate into urban metabolisms of building stocks: A framework for spatiotemporal material flow analysis and a case study. Environment and Planning B: Urban Analytics and City Science, 50(6), 1559–1576. https://doi.org/10.1177/23998083221140892

Kornmann, M., & Queisser, A. (2012). Service life of the building stock of Switzerland. Mauerwerk, 16(4), 210–215. https://doi.org/10.1002/dama.201290053

Kurvinen, A., Huovari, J., Lahtinen, M., Sen, T., & Saari, A. (2024). A dynamic model for estimating the long-term need for repairs and renovations in residential buildings. Building Research & Information, 52(5), 533–550. https://doi.org/10.1080/09613218.2024.2306371

Li, Q., Gummidi, S. R. B., Lanau, M., Yu, B., & Liu, G. (2022). Spatiotemporally Explicit Mapping of Built Environment Stocks Reveals Two Centuries of Urban Development in a Fairytale City, Odense, Denmark. Environmental Science & Technology, 56(22), 16369–16381. https://doi.org/10.1021/acs.est.2c04781

Liu, G., Xu, K., Zhang, X., & Zhang, G. (2014). Factors influencing the service lifespan of buildings: An improved hedonic model. Habitat International, 43, 274–282. https://doi.org/10.1016/j.habitatint.2014.04.009

Meijer, A., Arora, M., & Cheah, L. (2024). Material stock-service and circularity prospects of buildings in Singapore. Resources, Conservation and Recycling, 208, 107697. https://doi.org/10.1016/j.resconrec.2024.107697

Miatto, A., Schandl, H., Forlin, L., Ronzani, F., Borin, P., Giordano, A., & Tanikawa, H. (2019). A spatial analysis of material stock accumulation and demolition waste potential of buildings: A case study of Padua. Resources, Conservation and Recycling, 142, 245–256. https://doi.org/10.1016/j.resconrec.2018.12.011

Miatto, A., Schandl, H., & Tanikawa, H. (2017). How important are realistic building lifespan assumptions for material stock and demolition waste accounts? Resources, Conservation and Recycling, 122, 143–154. https://doi.org/10.1016/j.resconrec.2017.01.015

Müller, D., Cao, J., Kongar, E., Altonji, M., Weiner, P.-H., & Graedel, T. (2007). Service Lifetimes of Mineral End Uses.

O'Connor, J. (2004). Survey on actual service lives for North American buildings.

Østergaard, N., Thorsted, L., Miraglia, S., Birkved, M., Rasmussen, F. N., Birgisdóttir, H., Kalbar, P., & Georgiadis, S. (2018). Data Driven Quantification of the Temporal Scope of Building LCAs. Procedia CIRP, 25th CIRP Life Cycle Engineering (LCE) Conference, 30 April – 2 May 2018, Copenhagen, Denmark, 69, 224–229. https://doi.org/10.1016/j.procir.2017.11.057

Ran, H. (2016). The Investigation And Analysis On The Life-span Of Residential Buildings (Q. Dong & M. Guo, Eds.; Vol. 49, pp. 591–595). (WOS:000388287600130).

Reyna, J. L., & Chester, M. V. (2015). The Growth of Urban Building Stock: Unintended Lock-in and Embedded Environmental Effects. Journal of Industrial Ecology, 19(4), 524–537. https://doi.org/10.1111/jiec.12211

Rincón, L., Pérez, G., & Cabeza, L. F. (2013). Service life of the dwelling stock in Spain. The International Journal of Life Cycle Assessment, 18(5), 919–925. https://doi.org/10.1007/s11367-013-0552-x

Sanjo, K., Kayo, C., & Tonosaki, M. (2025). Lifetime functions and half-lives of residential building structures in Japan: A comparative study of wooden, non-wooden, and concrete block houses. Journal of Building Engineering, 108, 112916. https://doi.org/10.1016/j.jobe.2025.112916

Stolz, B., Li, J., & Wellinger, S. (2025). Urban Transformation Towards Premature Obsolescence Of Buildings. Enquiry, 22(1), 37–51. Scopus. https://doi.org/10.17831/enqarcc.v22i1.1261

Tanikawa, H., & Hashimoto, S. (2009). Urban stock over time: Spatial material stock analysis using 4d-GIS. Building Research & Information, 37(5–6), 483–502. https://doi.org/10.1080/09613210903169394

Wang, J., Zhang, Y., & Wang, Y. (2018). Environmental impacts of short building lifespans in China considering time value. Journal of Cleaner Production, 203, 696–707. https://doi.org/10.1016/j.jclepro.2018.08.314

Wang-Xu, M., Berglund-Brown, J., Clifford, B., & Ochsendorf, J. (2025). Leveling the playing field: Lifetimes of North American sports stadiums. Proceedings of IASS Annual Symposia, 2025(5), 1–12.

Xia, B., Xiao, J., Liu, G., Guan, X., Lu, Y., & Wang, Y. C. (2025). Exploiting vacant urban residential buildings to promote carbon neutrality in China. Nature Communications, 16(1), 7661. https://doi.org/10.1038/s41467-025-62879-4

Zhou, W., Moncaster, A., Reiner, D. M., & Guthrie, P. (2019). Estimating Lifetimes and Stock Turnover Dynamics of Urban Residential Buildings in China. Sustainability, 11(13), Article 13. https://doi.org/10.3390/su11133720

Zhou, W., O'Neill, E., Moncaster, A., Reiner, D. M., & Guthrie, P. (2022). Modelling Chinese Urban Residential Stock Turnover Uncertainties Using System Dynamics and Bayesian Statistical Inference. In Urban Infrastructuring: Reconfigurations, Transformations and Sustainability in the Global South: Part F2710 (pp. 221–240). Scopus. https://doi.org/10.1007/978-981-16-8352-7_14

1.4 Geographical coverage

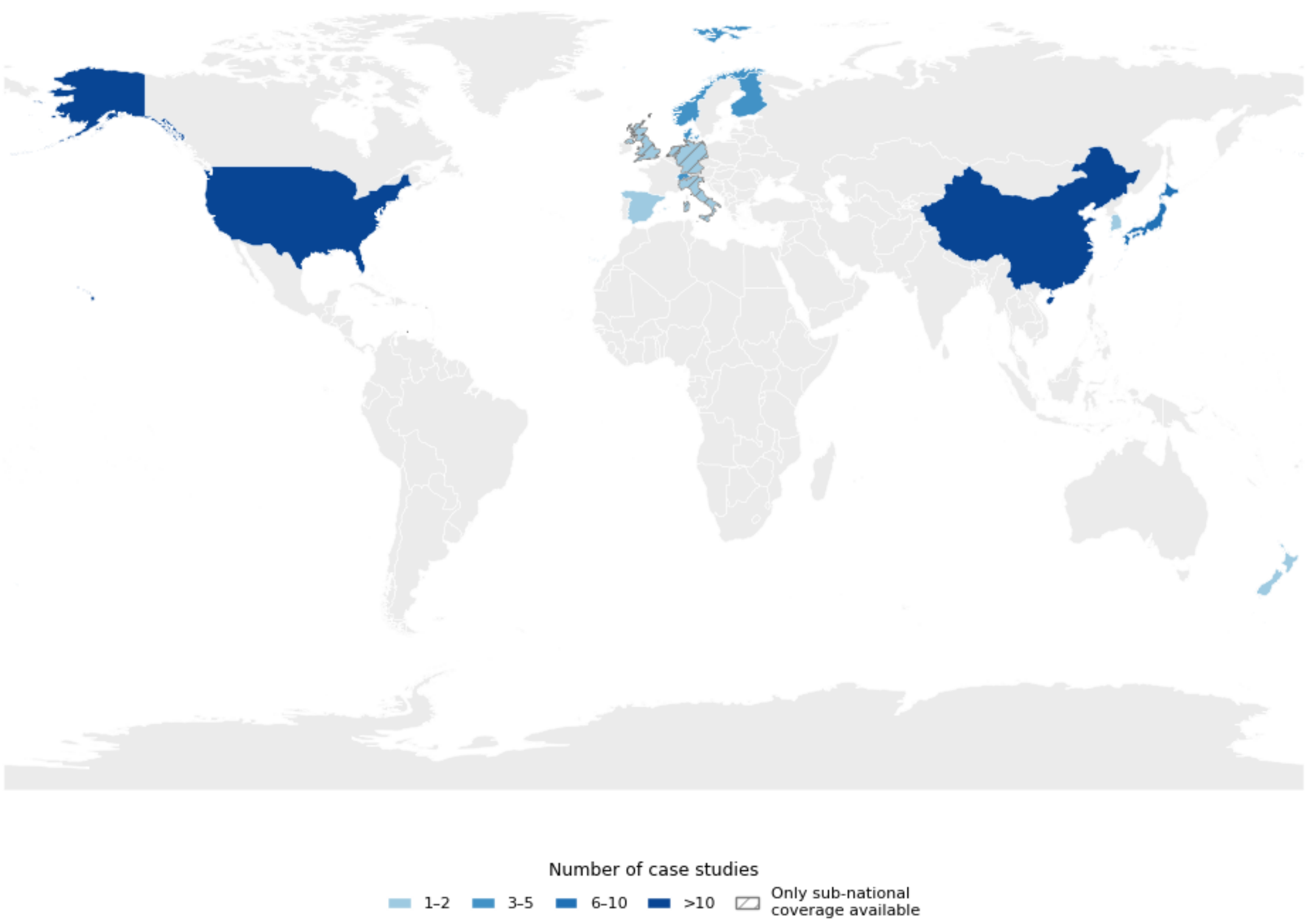

Figure S1: The geographical coverage of the identified 59 case studies. Several countries were featured with no national-level coverage.

2 Statistical analysis

2.1 Regression analysis clustering

The regression analysis considers clustering of observations employing the same data source. Such clustering is evident in the case of median lifetimes originating from the same case study (we identified 59 case studies within the 46 reviewed publications). Estimates within the same caste study inherently refer to the same building stock while differentiating, e.g., between use classes, spatial coverage areas like neighborhoods, structural material types.

In addition, several independent publications may use the same data source, making the resulting estimates correlated. This effect was considered by recording the data source of each study and flagging those that use the same core source of data. These estimates were clustered together, even though they still differed from one another due to various scope, assumptions and methodologies. Below we list the studies grouped together based on the same core source of data:

- Historical records of the city of Zurich – Aksözen et al. (2017a, 2017b)
- Americal Housing Survey – Aktas & Bilec (2012), Ianchenko et al. (2020)
- Danish register of buildings – Andersen & Negendahl (2023), Østergaard et al. (2018)
- Japanese statistics on constructed area and existing area – Daigo et al. (2017), Kayo & Tonosaki (2022), Sanjo et al. (2025)
- construction data from China Statistics Yearbook – Cai et al. (2015), Cai et al. (2025), Cao et al. (2019), Xia et al. (2025), Wang et al. (2018), Zhou et al. (2019, 2022)

2.2 Detailed results of the statistical analysis

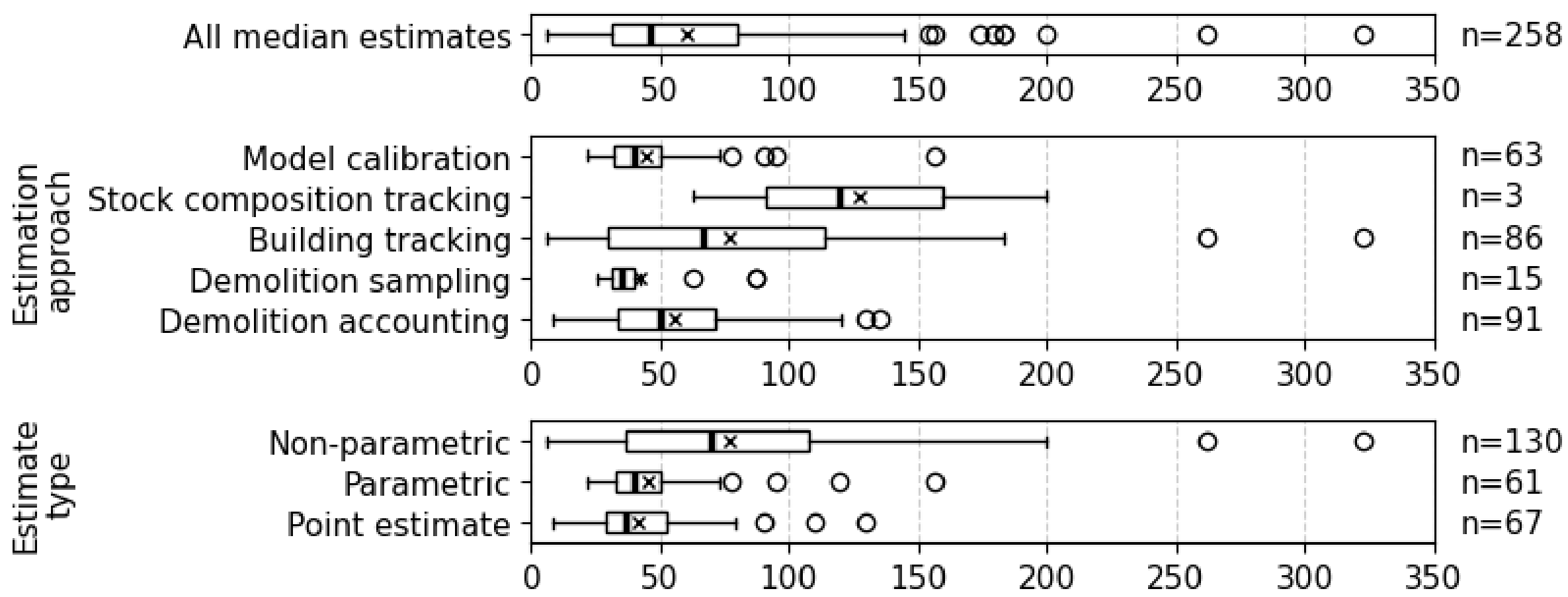

Figure S2: Statistical description of reported median estimates grouped by estimation approach and estimate type. The horizontal rectangle denotes the 25th-75th percentile range, the vertical thick line denotes the median, the cross denotes the mean.

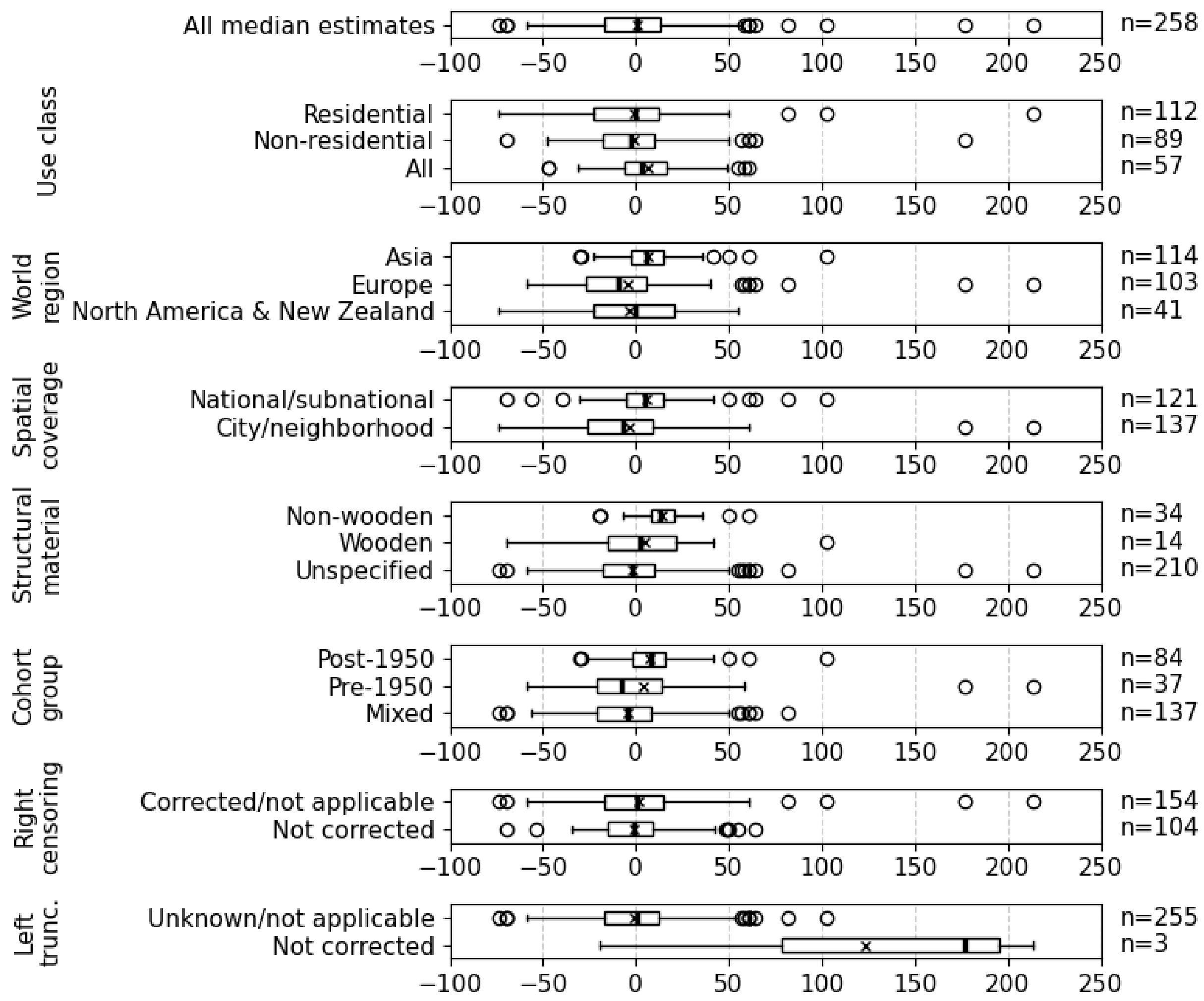

Figure S3: Regression model residuals for median lifetime estimates. The horizontal rectangle denotes the 25th-75th percentile range, the vertical thick line denotes the median, the cross denotes the mean.

Table S1: Predicted median values for chosen combinations of factors with the 95% confidence interval (CI) for average prediction and 95% prediction interval (PI) for individual prediction. The full list is available in Supporting Information SI2.

World region	Use class	Spatial coverage	Cohort group	Structural material	Right censoring	Predicted median [years]	95% CI [years]	95% PI [years]
Residential	Asia	National/ subnational	Post-1950	Non-wooden	Corrected/not applicable	23.36	[17.68, 30.87]	[10.73, 50.88]
Residential	Europe	National/ subnational	Post-1950	Non-wooden	Corrected/not applicable	57.52	[45.74, 72.35]	[26.85, 123.26]
Residential	North America & New Zealand	National/ subnational	Post-1950	Non-wooden	Corrected/not applicable	63.05	[47.66, 83.41]	[28.94, 137.38]
Residential	Asia	National/ subnational	Post-1950	Unspecified	Corrected/not applicable	27.44	[20.77, 36.25]	[12.6, 59.76]

Residential	Europe	National/subnational	Post-1950	Unspecified	Corrected/not applicable	67.56	[53.72, 84.97]	[31.53, 144.77]
Residential	North America & New Zealand	National/ subnational	Post-1950	Unspecified	Corrected/not applicable	74.05	[55.97, 97.97]	[33.98, 161.35]
Residential	Asia	National/ subnational	Post-1950	Wooden	Corrected/not applicable	53.81	[40.73, 71.1]	[24.71, 117.2]
Residential	Europe	National/ subnational	Post-1950	Wooden	Corrected/not applicable	132.5	[105.35, 166.65]	[61.84, 283.93]
Residential	North America & New Zealand	National/ subnational	Post-1950	Wooden	Corrected/not applicable	145.23	[109.77, 192.15]	[66.65, 316.45]
Non-residential	Asia	National/ subnational	Post-1950	Non-wooden	Corrected/not applicable	18.06	[13.67, 23.87]	[8.29, 39.34]
Non-residential	Europe	National/ subnational	Post-1950	Non-wooden	Corrected/not applicable	44.47	[35.36, 55.93]	[20.75, 95.3]
Non-residential	North America & New Zealand	National/ subnational	Post-1950	Non-wooden	Corrected/not applicable	48.75	[36.84, 64.49]	[22.37, 106.21]
Non-residential	Asia	National/ subnational	Post-1950	Unspecified	Corrected/not applicable	21.21	[16.06, 28.03]	[9.74, 46.2]
Non-residential	Europe	National/ subnational	Post-1950	Unspecified	Corrected/not applicable	52.23	[41.53, 65.69]	[24.38, 111.92]
Non-residential	North America & New Zealand	National/ subnational	Post-1950	Unspecified	Corrected/not applicable	57.25	[43.27, 75.74]	[26.27, 124.75]
Non-residential	Asia	National/ subnational	Post-1950	Wooden	Corrected/not applicable	41.6	[31.49, 54.97]	[19.1, 90.61]
Non-residential	Europe	National/ subnational	Post-1950	Wooden	Corrected/not applicable	102.44	[81.45, 128.85]	[47.81, 219.52]
Non-residential	North America & New Zealand	National/ subnational	Post-1950	Wooden	Corrected/not applicable	112.28	[84.86, 148.56]	[51.53, 244.66]
